# *APOBR* Is Downregulated in EBV+ Tonsils of Children with Obstructive Sleep-Disordered Breathing

**DOI:** 10.3390/genes15101324

**Published:** 2024-10-14

**Authors:** Regie Lyn P. Santos-Cortez, Helen Z. Gomez, Christina L. Elling, Landen Mayher, Obinna R. Diala, Colin Gardner, Kiera Willford, Valerie C. Zamora, Ashley Agyepong, Nam K. Lee, Katherine K. Green, Owen A. Darr, Todd M. Wine, Christian R. Francom, Eric D. Larson, Sarah A. Gitomer, Amy E. Schell, Daniel N. Frank, Norman R. Friedman, Brian W. Herrmann

**Affiliations:** 1 Department of Otolaryngology-Head and Neck Surgery, School of Medicine, University of Colorado Anschutz Medical Campus, Aurora, CO 80045, USA; helen.gomez@cuanschutz.edu (H.Z.G.); christina.elling@cuanschutz.edu (C.L.E.); landen.mayher@cuanschutz.edu (L.M.); colin.gardner@cuanschutz.edu (C.G.); nam.lee@cuanschutz.edu (N.K.L.); katherine.green@cuanschutz.edu (K.K.G.); owen.darr@childrenscolorado.org (O.A.D.); todd.wine@childrenscolorado.org (T.M.W.); christian.francom@childrenscolorado.org (C.R.F.); larsoned@upenn.edu (E.D.L.); sarah.gitomer@childrenscolorado.org (S.A.G.); amy.schell@cuanschutz.edu (A.E.S.); norman.friedman@childrenscolorado.org (N.R.F.); brian.herrmann@childrenscolorado.org (B.W.H.); 2 Department of Pediatric Otolaryngology, Children’s Hospital Colorado, Aurora, CO 80045, USA; dialao@vcu.edu; 3 Pathways in Genomic Research for Undergraduate Experiences (PATH-GREU) Program, University of Colorado Denver|Anschutz Medical Campus, Aurora, CO 80045, USA; kwillfor@msudenver.edu; 4 Colorado Research Experiences (CORE) Program, University of Colorado Anschutz Medical Campus, Aurora, CO 80045, USA; valeriezamora007@gmail.com (V.C.Z.); ashleyagyepong@gmail.com (A.A.); 5 Department of Basic and Translational Science, School of Dental Medicine, University of Pennsylvania, Philadelphia, PA 19104, USA; 6 Division of Infectious Diseases, Department of Medicine, School of Medicine, University of Colorado Anschutz Medical Campus, Aurora, CO 80045, USA; daniel.frank@cuanschutz.edu

**Keywords:** *APOBR*, bulk mRNA sequencing, continuous positive airway pressure, CPAP, EBV, Epstein–Barr virus, obstructive sleep-disordered breathing, RNA-seq, sleep apnea, tonsil

## Abstract

***Background:*** Obstructive sleep-disordered breathing (oSDB) is a heterogeneous phenotype that is increasing in prevalence worldwide and has many potential comorbidities that could severely affect quality of life. There is a need to identify biomarkers for oSDB and its comorbidities to improve clinical management, particularly in children. ***Methods:*** We performed bulk mRNA-sequencing, differential expression analysis, and qPCR replication of selected differentially expressed genes (DEGs) using RNA samples extracted from tonsils of children with oSDB. Two variables were used as classifier, namely, detection of Epstein–Barr virus (EBV) in tonsils and need for continuous positive airway pressure (CPAP) treatment. Standard statistical tests were used to determine associations across clinical, EBV, and DEG variables. ***Results:*** Nineteen genes were dysregulated in tonsils that are EBV+ or from children needing CPAP. Of these genes, *APOBR* was downregulated in both EBV+ and CPAP+ tonsils, and this downregulation was replicated by qPCR in an independent set of pediatric samples. In the tonsils of adult patients with oSDB, *APOBR* was positively correlated with age, and potentially with diastolic blood pressure. ***Conclusions:*** Taken together, *APOBR* and DEGs in tonsillar tissues may be useful as potential biomarkers of oSDB severity and comorbidity across the lifespan, with *APOBR* levels being dependent on latent EBV infection.

## 1. Introduction

Obstructive sleep-disordered breathing (oSDB) is an ill-defined, heterogeneous phenotype comprised of a spectrum of sleep-related disorders, spanning from habitual snoring without hypoxic episodes to documented obstructive sleep apnea (OSA) or hypoventilation [1,2]. If the intermittent hypoxia and sleep disruption in moderate-to-severe oSDB or OSA are left untreated, comorbidities that include neurocognitive and behavioral issues, cardiovascular and lung diseases, insulin resistance, etc., may worsen or ensue [2,3].

Currently, the diagnosis of oSDB is mostly clinical. oSDB is broadly defined in children as having fragmented sleep and behavioral issues due to upper airway obstruction [4,5,6]. However, only a small proportion of children with oSDB are prescribed sleep study or polysomnography (PSG) to obtain a definitive diagnosis of OSA. Instead, depending on symptom severity and comorbid conditions, the majority of children with oSDB undergo surgical removal of tonsils (tonsillectomy) as first-line treatment [5,6,7]. Resolution of oSDB symptoms after tonsillectomy is variable in children (10–79%), with higher risk of symptom recurrence in children who are older and have more severe oSDB or obesity, among others [6,7]. For children who cannot undergo surgery or remain symptomatic after surgery, continuous positive airway pressure (CPAP) is the next option for treatment. In previous studies, it was shown that children who are older and have more severe oSDB or obesity are at risk of poor adherence to CPAP as treatment [7,8].

In contrast, the diagnosis and treatment of OSA in adults are mostly based on sleep study findings, with CPAP as the first option for treatment [9]. Whether to prescribe CPAP for treatment of comorbid hypertension in adults is controversial. There are more surgical options for severe OSA in adults, although tonsillectomy and removal of pharyngeal tissues via uvulopalatopharyngoplasty are still the most common techniques. Drug-induced sleep endoscopy has been applied more frequently as a preoperative evaluation tool for hypoglossal nerve stimulation to treat CPAP-intolerant moderate-to-severe OSA in adults [10,11]. Currently, OSA endotypes—high critical closing pressure, low arousal threshold, high loop gain, and impaired responsiveness of upper-airway muscle—are being extrapolated from PSG data and hailed as more sensitive measures for classifying OSA subtypes and targeting novel therapies [12,13]. Unfortunately, not all adult or pediatric patients with oSDB have available pre- and post-treatment PSG data, which is partly due to the time, cost, and effort involved in performing PSG, thus remaining prohibitive for some patients.

Because of the potential morbidity arising from oSDB at any age, as well as the lack of diagnostic options that clearly define oSDB sub-phenotypes that will guide treatment, there is a great need to identify biomarkers for the diagnosis and response to treatment of oSDB and its comorbidities. The use of transcriptomics has great potential in identifying novel biomarkers in relevant tissues. However most transcriptomic research for OSA has used blood samples rather than tonsil tissues, the latter being the oSDB-relevant tissue type in the upper airway, particularly in children. Tonsillar transcriptomic studies, whether in children or adults, remain sparse in the literature. One microarray study (*n* = 88) identified 22 pathways enriched in palatine tonsils from children with oSDB; proliferation was the top pathway, leading to the differentially expressed gene (DEG) *PSPH* as a putative target for programmed cell death in order to address tonsillar hypertrophy [14]. Another mRNA-seq study (*n* = 52) examined tonsils of atopic and non-atopic subjects rather than oSDB [15].

It was previously reported that tonsillar hypertrophy in children as indicated by higher Brodsky scores, which are based on the degree of tonsil enlargement impinging upon the upper airway space, were associated with latent (asymptomatic) infection by Epstein–Barr virus (EBV) [16]. Here, we report our findings from the tonsillar transcriptomes of children with oSDB, according to two variables: (1) detection of EBV in tonsil tissues and (2) recommendation for CPAP as treatment. We subsequently identified DEGs that were part of signaling pathways and that may contribute to oSDB pathology. Moreover, one gene, *APOBR*, had significantly decreased expression in tonsils positive for EBV and in children requiring CPAP.

## 2. Materials and Methods

This study was approved by the Colorado Multiple Institutional Review Board (protocol 17-0714). Informed consent was obtained from adult patients and from parents of children who participated in this study. Child assent was also documented for children who were at least seven years old.

Children 6 months or older were recruited into this study if undergoing tonsillectomy and providing palatine tonsil samples. All children tested negative for SARS-CoV-2 PCR of nasopharyngeal swabs prior to surgery. Likewise, adult patients with oSDB were recruited as a potential replication cohort. From the clinical records, variable data were collected from both children and adult patients (Table S1). When sleep study was performed, data on sleep parameters were also extracted per patient (Table S2). Standard statistical analyses (chi-square or Fisher exact, *t*-test, Kruskal, and Pearson or Spearman correlation) were performed to determine associations between clinical variables, while analyzing pediatric and adult data separately.

DNA and RNA samples were isolated from tonsil tissues using the Qiagen AllPrep DNA/RNA Micro Kit. DNA samples were tested for EBV using synthetically derived EBV Primer ASR (Qiagen, Germantown, MD, USA), which amplifies a 97-base pair region of the EBNA-1 gene of the EBV genome. PCR was performed under standard conditions and gel electrophoresis was used to determine if the sample was positive for EBV. A patient sample that was previously determined to be positive for EBV was used as positive control.

RNA samples isolated from sixteen pediatric tonsils were submitted to the Genomics and Microarray Core at the University of Colorado Anschutz Medical Campus and passed quality control (median RIN 7, minimum RIN 6) for bulk mRNA-sequencing using the Illumina Poly A Selected Total RNA Library and a NovaSEQ 6000 instrument (Illumina, San Diego, CA, USA). The generated FASTQ files were trimmed using BBDuk39.10 [17] and mapped to Gencode GRCh38.p13 using Salmon v1.4.0 [18]. DESeq2 1.44.0 [19] was used to determine differential gene expression based on (a) EBV-positive vs. EBV-negative, and (b) recommended for CPAP use vs. not. Differential expression analyses were corrected for age at surgery.

Using the 19 differentially expressed genes (DEGs; Table 1) as input, network analysis was performed using NetworkAnalyst (https://www.networkanalyst.ca/ access on 10 October 2024) [20]. To determine interactions among the DEGs, transcription factor-miRNA coregulatory interaction data from the RegNetwork [21] repository was selected among the Gene Regulatory Networks databases.

To validate RNA-seq findings, qPCR of *APOBR* was performed using tonsil RNA samples which were collected from 32 children and 14 adult patients with oSDB and which were not previously submitted for bulk mRNA-sequencing. Primers were designed to amplify exons 3 and 4 of *APOBR* as follows: forward primer 5′-CTGGGACACGGGTTTGGC-3′; reverse primer 5′-CTCAGTCACTGGGGCTTAGG-3′. EBV was used as the classifier variable to determine fold change, and difference in expression level (deltaCq) was analyzed using t-test. *GAPDH* was used as the control gene. Additionally, using CPAP use as a classifier, we performed qPCR of *TRIM39*, *WNT7A*, and *ACTB* (control) using the following primers: *TRIM39* forward primer 5′-CAATTTCCCACACCCACCG-3′; *TRIM39* reverse primer 5′-CCCAAGGTGAGCAGCATTTT-3′; *WNT7A* forward primer 5′-TTCGGGAAGGAGCTCAAAG-3′; *TRIM39* reverse primer 5′- TGTGGTCCAGCACGTCTT-3′.

## 3. Results

### 3.1. Clinical Profile of the Pediatric oSDB Cohort

We collected clinical data and tonsil tissues from 119 children, of whom 113 (95%) were diagnosed with oSDB and 6 (5%) had chronic tonsillopharyngitis, tonsillar hypertrophy, and/or tonsillolith without oSDB. Ethnicity was 54% non-Hispanic White, 28% Latino, and 12% mixed. The cohort had an almost equal male/female ratio. Females had lower diastolic blood pressure (*p* = 0.04).

The average age at surgery was 7.5 years (median 6.1, range 1.9–18.5). The average body mass index (BMI) was 19.9 (median 16.7, range 13.2–68.6), with the mean BMI for age percentile at 66.4 (median 66.6, range 1–99.8), indicating healthy weight for the majority of children. Average blood pressure was 108/68 (maximum: systolic 143, diastolic 99). As expected, age was positively correlated with anthropometric traits height (*p* = 2.2 × 10^−16^), weight (*p* = 2.2 × 10^−16^), and BMI (*p* = 1.9 × 10^−5^), as well as blood pressure (systolic *p* = 0.002; diastolic *p* = 0.02). Around half of the children (*n* = 58) had data available from the Pediatric Sleep Questionnaire, which documents parental concern for oSDB-related symptoms (Tables S1 and S3) [4,7]. Among the questionnaire variables, only history of previous chronic or recurrent tonsillitis (present in 10 out of 58, or 17.2%) was associated with age (*p* = 0.043).

Regarding the known comorbidities of oSDB, higher systolic blood pressure in children was associated with poor school performance based on the sleep questionnaire (*p* = 4.6 × 10^−7^). This finding might suggest that the effects of oSDB on the peripheral vasculature and cognitive function are detected in childhood. In a recent clinical trial of adenotonsillectomy vs. watchful waiting for children with mild oSDB, cognition did not improve 12 months post-surgery, but behavioral issues, sleepiness, and quality of life improved and blood pressure decreased [22].

### 3.2. Sleep Study Results in the Pediatric oSDB Cohort

Of the 119 recruited children, 24 (20%) underwent diagnostic (*n* = 18) or split night (*n* = 6) sleep study or polysomnography (PSG). The lower proportion of children undergoing sleep study follows current Clinical Practice Guidelines [4,5,6] wherein only children with oSDB who are <2 years old or those with predisposing conditions (e.g., obesity, craniofacial abnormalities) are recommended for PSG. Female children with sleep test findings had a higher mean heart rate during the total sleep time/TST (*p* = 0.001) and higher oxygen desaturation index (*p* = 0.047) than in males.

Of the 24 children with PSG results, 13 (54%) had a BMI for age at >85th percentile, 4 had syndromes (Turner, Down, VACTERL), and the rest were highly symptomatic based on the sleep questionnaire variables. For the commonly used measures for severity of OSA, the mean Apnea–Hypopnea Index (AHI) was 10.3, mean obstructive (O)AHI was 11.2 (indicating severe OSA at OAHI > 10), mean value for Central (C)AHI was 0.93, and Central Apnea Index (CAI) was 1.1. Among the PSG parameters, age was positively correlated with rapid eye movement (REM) latency (*p* = 0.02) and %sleep time with arterial oxygen saturation SpO2 < 90 (*p* = 0.02). On the other hand, age was negatively correlated with %sleep time at REM (*p* = 0.0002), REM events time (*p* = 0.004), CAI (*p* = 0.008), and CAHI (*p* = 0.03). Overall, these correlations in our sleep study data in this subset of children suggest worse oSDB in older children.

When compared with children diagnosed with tonsillitis but no oSDB, children with oSDB were younger (*p* = 0.02), shorter in height (*p* = 0.0097), and lighter in weight (*p* = 0.045). Increased OAHI, which is used to classify severity of OSA, was not associated with age and anthropometric variables. Instead, higher BMI was associated with snoring (*p* = 0.003), was positively correlated with systolic blood pressure (*p* = 0.0006), and with an increase with the following sleep study parameters: %sleep time at SpO2 < 90 (*p* = 0.028), AHI (*p* = 0.016), and supine OAHI (*p* = 0.0003). In contrast, a history of recurrent or chronic tonsillitis was associated with older age (*p* = 0.043); taller height (*p* = 0.007); longer TST (*p* = 0.02); non-supine event time (*p* = 0.0003); shorter REM latency (*p* = 0.002), stage N1 (*p* = 0.04), and N3 sleep (*p* = 0.048); an increased minimum SpO2% TST (*p* = 0.008); lower mean heart rate TST (*p* = 0.03), %sleep time with SpO2 < 90 (*p* = 0.02), and oxygen desaturation index (*p* = 0.01); and lower AHI (*p* = 0.006) and zero OAHI (*p* = 0.002). Taken together, these measures indicate better sleep in children with both oSDB diagnosis and a known history of recurrent or chronic tonsillitis; conversely, those with oSDB only and higher BMI had worse sleep data.

Among the other diseases identified in the cohort, occurrence of allergies (39% of cohort) and asthma (13% of cohort) had various associations with exposure variables and sleep parameters, namely: for allergies—taller height, exposure to parental or caregiver tobacco smoke, higher mean heart rate, and lower mean SpO2% (whether for REM sleep, non-REM, or TST); for asthma—older age, taller height, and less periodic breathing (as % of sleep time). From the questionnaire variables, behavioral issues (e.g., impulsivity, inattentiveness) were similarly associated with longer stage N2 sleep, lower oxygen desaturation index, no periodic breathing, lower supine OAHI and non-REM events OAHI, and overall better sleep with lower CAI, CAHI, AHI, and OAHI.

Only four children (3.4%) were recommended for CPAP pre- and post-tonsillectomy. All sleep studies for the four children were conducted at least 2 weeks and up to 7 months prior to surgery. Better sleep efficiency was associated with CPAP use (*p* = 0.03). However, no sleep tests were performed after surgery.

### 3.3. EBV Prevalence in Pediatric Tonsils

Of the 97 DNA samples extracted from pediatric tonsils that were tested for EBV, 46 (47.4%) or almost half of samples were EBV-positive. This EBV prevalence rate in our cohort is similar to previous reports in pediatric tonsils (40–53%) [16,23,24]. Among the demographic and clinical variables, EBV positivity was associated with ethnicity (*p* = 0.002), with tonsillar EBV prevalence of 32.7% among White children, 75% among Latinos, and 50% with another ethnicity. Among the sleep test parameters (*n* = 19), a decrease in the % of time with CO_2_ > 45 mmHg was observed in children with EBV+ tonsils (*p* = 0.047).

### 3.4. Differentially Expressed Genes from Bulk mRNA-Seq of Pediatric Tonsils

To generate hypotheses on the clinical relevance of EBV in oSDB, we selected tonsil tissues from 16 children for bulk mRNA-sequencing. For the children who provided the 16 tonsil RNA samples, the average age was 8.3 years, average BMI 24, and average BMI percentile for age was 75.6. Females comprised 44%, 56% of the 16 children had allergies, and 25% had a history of chronic/recurrent tonsillitis. Three children were recommended for CPAP due to OAHI ≥ 18 or BMI for age ≥99th percentile. The demographic and clinical variables according to EBV-positive or negative samples are reported in Table S3.

**Figure 1:**
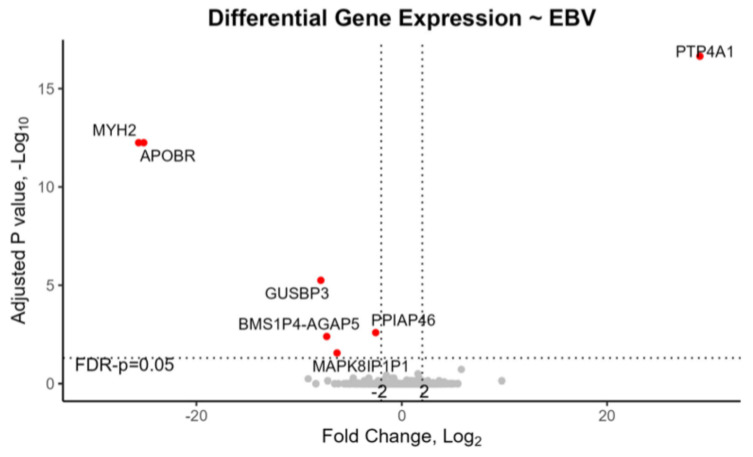
Volcano plot for differentially expressed genes in EBV-positive versus EBV-negative pediatric tonsils.

**Figure 2:**
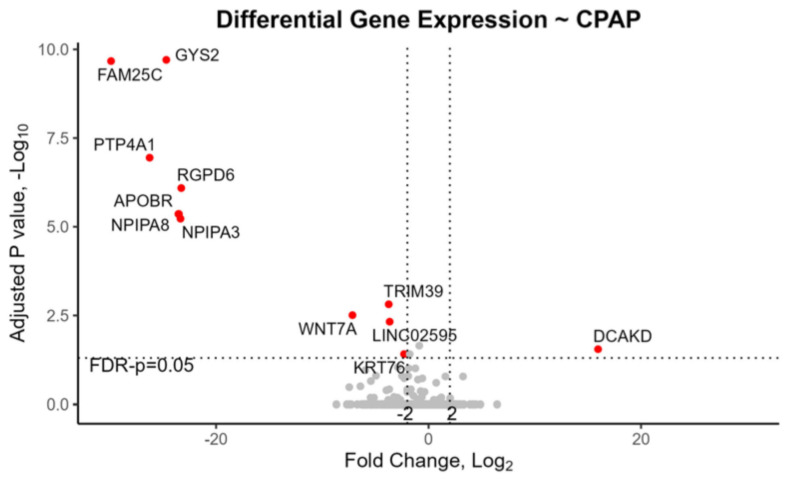
Volcano plot for differentially expressed genes in the tonsils of children who were prescribed with CPAP versus not. Two genes *GK5* and *TCF7L2* are not included graphically because of log-fold changes that have absolute values less than 2.

In EBV+ tonsils with mRNA-seq data (7 out of 16, or 43.8%), two genes, namely, *APOBR* and *MYH2*, plus four pseudogenes *GUSBP3*, *PPIAP46*, *BMS1P4-AGAP5*, and *MAPK8IP1P1*, were downregulated while one gene, *PTP4A1*, was upregulated (Table 1, Figure 1). Moreover, in the tonsils of children recommended for CPAP, one gene *DKACD* was upregulated, and thirteen transcripts were downregulated. Notably APOBR was significantly downregulated both in EBV+ tonsils and in children requiring CPAP (Table 1, Figure 1 and Figure 2). *PTP4A1* was downregulated in the tonsils of children recommended for CPAP but was upregulated in the opposite direction for EBV+ tonsils. Of these genes, *TRIM39*, *LINC02595*, *DKACD*, and *TCF7L2* were previously associated with OSA or insomnia in genome-wide association studies (GWAS) [25,26]. Loci for the other genes—*PTP4A1*, *APOBR*, *BMS1P4-AGAP5*, *MAPK8IP1P1*, *GYS2*, *FAM25C*, and *GK5*–were formerly associated with traits involved with comorbidities of OSA, i.e., BMI, weight, triglycerides, blood pressure, stroke, brain volume, cortical thickness, mood, insulin, and diabetes [27].

### 3.5. Network Analysis Using DEGs

Network analysis revealed that 12 DEGs (Table 1) among 244 genes and miRNAs are connected through protein–protein interactions (Figure S1) from the STRING database and transcription factor-miRNA co-regulatory interactions from the RegNetwork. The gene network was significantly enriched with 51 KEGG pathways (FDR-*p* < 0.05; Table S4) that predominantly consisted of signaling pathways, such as Wnt, cancer, Notch, thyroid hormone, TGF-β, glucagon, as well as adherens junction and viral infections (e.g., HTLV-1, herpesvirus, EBV, measles). Only viral carcinogenesis overlapped with findings in previous studies [25]; thus, our DEG and pathway findings are mostly novel.

### 3.6. Replication of EBV-APOBR Association in Pediatric Tonsils

From our bulk mRNA-seq data, *APOBR* was downregulated in both EBV+ tonsils and in children requiring CPAP due to oSDB (Table 1). To validate mRNA-seq findings, an independent subset of 32 pediatric tonsils were selected for qPCR of *APOBR.* None of these tonsils overlapped with those used for bulk mRNA-sequencing, and 20 (62.5%) of these tonsils were EBV+. qPCR revealed downregulation of *APOBR* in this independent dataset, with a 0.31-fold change or 69% reduction in *APOBR* expression in EBV+ compared to EBV- tonsils (*p* = 0.026; Table 2).

On the other hand, qPCR of *TRIM39* and *WNT7A* on 18 pediatric tonsils did not yield significant results according to CPAP use (Table 2).

### 3.7. APOBR Expression in Tonsils from Adult Patients with oSDB

We also collected clinical data and tonsil tissues from 19 adults with oSDB. Eight (47%) had preoperative PSG data [11,28,29,30]. All adult patients required CPAP prior to surgery. The average age at surgery was 34.6 years (range 18–52). Males comprised 59% and were older than females (mean age for males was 40 years vs. 27 years in females), 65% were non-Hispanic White, and 29% Latino. Of note, all ten male patients were White, while all five Latino patients were female and had a family history of diabetes. The average BMI was 30 (median 29; range 24.9–35), indicating overweight or obesity for the majority of recruited adult patients. A family history of hypertension (*p* = 0.01) was associated with higher BMI. Average blood pressure was 129/84 (maximum: systolic 189; diastolic 100). Systolic blood pressure increased with age (*p* = 0.037) and was higher in males (*p* = 0.02). In those with sleep test results, systolic blood pressure increased with a lower minimum SpO2 (i.e., increased hypoxic state; *p* = 0.03).

DNA and RNA samples from 14 adult patients with oSDB were available for study. Thirteen out of the fourteen tonsils were positive for EBV, resulting in a 93% EBV+ prevalence rate in adult tonsils. This prevalence rate is consistent with reported prevalence of EBV in ≥70% of adult tonsils [31,32] but prevented testing of a potential association between EBV and *APOBR* expression in adult tonsils. In addition, qPCR for *APOBR* expression was performed using tonsillar RNA samples from the 14 adult patients. *APOBR* levels were positively correlated with age (+0.56, *p* = 0.04). In the adult patients with oSDB, there was a trend towards a positive correlation between increased *APOBR* expression and diastolic blood pressure (+0.51, *p =* 0.064).

## 4. Discussion

To summarize, in our pediatric cohort, worse sleep was associated with older age, higher BMI, and allergies. Increased BMI was correlated with higher systolic blood pressure, which in turn was strongly associated with poor school performance. These associations are consistent with findings from longitudinal studies [33]. However, many of the demographic, anthropometric, clinical, and PSG variables are highly correlated, and the identified associations may not be informative for treatment decisions. Because PSG has a limited indication in children with oSDB and remains prohibitive even for some adult patients, alternative cheaper and user-friendly screening methods such as biomarker testing are needed for the diagnosis and subclassification of oSDB, which could facilitate profile-guided treatment.

In the tonsils of our pediatric and adult patients with oSDB, the prevalence of EBV detection was 47.4% and 93%, respectively. Within the head and neck, EBV is most known for causing acute infectious mononucleosis in adolescents, with reports of acute airway obstruction requiring tonsillectomy or chronic daytime sleepiness/sleep disorder [34,35,36,37,38,39]. Histologically, tonsils acutely infected with EBV had follicular hyperplasia, expanded interfollicular zones with lymphoid cell hyperplasia, necrotic cells near the tonsillar crypts, and lymphoid cells in crypt epithelium [37]. An overwhelming majority of infected cells are B lymphocytes, plus around 9% T cells, which are mostly CD8+. However, EBV infection is often asymptomatic at any age. When EBV becomes latent or persists silently in B lymphocytes, it affects transcription of adhesion molecules, growth factors, and inflammatory cytokines, which may in turn contribute to several neoplastic and autoimmune diseases, i.e., lymphoma, nasopharyngeal carcinoma, multiple sclerosis, and chronic fatigue syndrome. The innate immune response to latent EBV is partly modulated by activated macrophages M1 (CD68+) or M2 (CD163+), which are induced by and result in the secretion of pro- vs. anti-inflammatory cytokines, respectively [40]. However, the effect of EBV latency in tonsils within the context of oSDB is not well-studied. In one previous study, EBV was associated with tonsillar hypertrophy, as indicated by higher Brodsky scores in children [16]. Whether EBV exacerbates or directly contributes to oSDB pathology is unknown.

Our mRNA-seq study revealed multiple DEGs involved in signaling pathways within EBV+ pediatric tonsils, and also in tonsils of children requiring CPAP (Table 1, Figure 1 and Figure 2, Table S4, Figure S1). Among the DEGs we identified, *APOBR* was downregulated in EBV+ tonsils in both the mRNA-seq study and in the qPCR of an independent set of tonsil samples, providing replication (Table 1 and Table 2, Figure 1). Additionally, *APOBR* was downregulated (Table 1, Figure 2) in tonsils of children requiring postoperative CPAP, i.e., children with severe enough oSDB that remain symptomatic after tonsillectomy. *APOBR* encodes apolipoprotein B receptor, an intracellular macrophage receptor that binds to apolipoprotein B48 and facilitates uptake of triglyceride-rich lipoproteins [41]. Previous immunohistochemistry localized APOBR protein to macrophages and foam cells within atherosclerotic plaques [41]. It was hypothesized then that APOBR provides essential lipids to reticuloendothelial cells including macrophages, but overloading of its saturation kinetics might lead to foam cell formation and increased risk for cardiovascular disease [41].

In the Human Protein Atlas [42], among the tissues involved with oSDB and its comorbidities, APOBR is localized to cells in various degrees within the following: tonsil–non-germinal center (medium) and squamous epithelium (low); adipocytes (low); soft tissue–fibroblasts (medium) and peripheral nerve (medium); lung macrophages (high); cerebral cortex–endothelial (medium), glial cells (low), and neuropil (low). Single-cell RNA expression of *APOBR* is primarily detected in macrophages of the lung, muscle, tongue, esophagus, and blood vessels; macrophages and Schwann cells of adipose tissue; brain microglia; bronchial club cells; lymph node T cells; and serous glandular cells in salivary glands. There is also evidence of *APOBR* enrichment in adipose or lung neutrophils and thyroid macrophages and T cells. Few associations have been identified for *APOBR*, e.g., DNA methylation locus for obesity, GWAS loci for allergy and pneumonia, and transcript upregulation in *Streptococcus pneumoniae* infection (pneumonia, keratitis, and sepsis) [43,44,45,46]. Not much is known about the effects of cellular dysfunction due to *APOBR* downregulation. From the International Mouse Phenotyping Consortium [47], the *Apobr*-knockout mouse has no known significant phenotype, whether for mortality, growth, respiratory, fat metabolism, neurobehavioral, cardiovascular, or immune systems. Given the wide expanse of cell- and tissue-specific expression of *APOBR*, we may surmise that the long-term effects of *APOBR* downregulation in children with severe oSDB and EBV+ tonsils are not only localized to the oropharynx and might facilitate the development of comorbidities in other tissues including hypertension. Interestingly, in our adult patients, tonsillar *APOBR* was positively correlated with age and diastolic blood pressure, suggesting a non-linear relationship among age, blood pressure, and *APOBR* expression levels in the tonsil within the context of oSDB or OSA. 

Future studies may include a larger sample size of paired tonsil and blood samples for transcriptomic study with a wider expanse of age groups and with longitudinal data from blood transcriptome and blood pressure measurements. We will also need to validate protein levels in tonsil tissues, e.g., using proteomics. Unfortunately, viruses were detected from our bulk mRNA-seq data only at very low levels; therefore, at this time, viral detection is still better performed with PCR techniques until meta-transcriptomic technologies improve. Based on our key findings and what is known in the literature, we hypothesize that *APOBR* expression may contribute to oSDB pathology initially through its actions within the macrophages of upper-airway tissues and blood vessels, and might be modulated by low-level tissue inflammation, e.g., due to latent EBV infection in the tonsil.

## 5. Conclusions

To conclude, we identified *APOBR* and several DEGs in tonsillar tissues as potential biomarkers of oSDB severity and comorbidity across the lifespan, with *APOBR* levels being dependent on latent EBV infection.

## Figures and Tables

**Table 1:** Differentially expressed genes in EBV+ tonsils or in children recommended for CPAP due to oSDB.

Gene	Fold Change	False Discovery Rate Adjusted *p*-Value
**A. EBV+**		
***PTP4A1*** ^1^	**+29.04**	**2.25 × 10^−17^**
***APOBR*** ^1^	**−25.14**	**5.63 × 10^−13^**
*MYH2* ^1^	−25.63	5.63 × 10^−13^
*GUSBP3* ^1,2^	−7.88	5.62 × 10^−6^
*PPIAP46* ^2^	−2.54	0.0026
*BMS1P4-AGAP5* ^2^	−7.31	0.004
*MAPK8IP1P1* ^2^	−6.31	0.028
**B. CPAP**		
*GYS2* ^1^	−24.69	1.97 × 10^−10^
*FAM25C*	−29.89	2.14 × 10^−10^
***PTP4A1*** ^1^	**−26.26**	**1.13 × 10^−7^**
*RGPD6* ^1^	−23.28	8.11 × 10^−7^
***APOBR* ** ^1^	**−23.50**	**4.36 × 10^−6^**
*NPIPA8*	−23.55	4.36 × 10^−6^
*NPIPA3*	−23.36	5.92 × 10^−6^
*TRIM39* ^1^	−3.75	0.0015
*WNT7A* ^1^	−7.17	0.0031
*LINC02595* ^2^	−3.66	0.0047
*GK5* ^1^	−0.88	0.022
*DCAKD* ^1^	+15.96	0.028
*KRT76* ^1^	−2.31	0.039
*TCF7L2* ^1^	−1.78	0.039

^1^ Twelve genes or pseudogenes included in the interaction network (Figure S1). ^2^ Pseudogenes, except for *LINC02595,* which is a long intergenic non-protein coding RNA.

**Table 2:** qPCR validation results for three differentially expressed genes using pediatric tonsils.

Gene	*n*	Classifier	Log_2_ Fold-Change	*p*-Value
*APOBR*	32	EBV±	0.31	0.026
*TRIM39*	18	CPAP±	0.95	0.83
*WNT7A*	18	CPAP±	0.58	0.45

## Data Availability

Generated bulk mRNA-sequence data were uploaded to the Gene Expression Omnibus data repository (GSE274855).

## Supplementary Materials

The following supporting information can be downloaded at: https://www.mdpi.com/article/10.3390/genes15101324/s1, Table S1: Clinical variables; Table S2: Sleep study or polysomnography (PSG) parameters; Table S3: Characteristics of 16 children with oSDB and mRNA-seq data according to EBV±; Table S4: Kyoto Encyclopedia of Genes and Genomes (KEGG) pathways that are enriched within the gene network connecting 12 differentially expressed genes in pediatric tonsils; Figure S1: Kyoto Encyclopedia of Genes and Genomes (KEGG) pathways that are enriched within the gene network connecting 12 differentially expressed genes in pediatric tonsils.
