# Supplementary material for: APOBR Is Downregulated in EBV+ Tonsils of Children with Obstructive Sleep-Disordered Breathing
Source: Genes (Basel). 2024 Oct 14;15(10):1324. doi: 10.3390/genes15101324 (PMC11507010; doi:10.3390/genes15101324)
Supplement: Supplementary file 1 [file genes-15-01324-s001.zip › genes-3030838-supplementary.pdf]

Table S1. Clinical variables[1]

Demographic	Anthropometric /blood pressure	Medical history	Family history	Personal history
▪ Age at surgery<br>▪ Age at post-op follow-up/PSG<br>▪ Sex<br>▪ Ethnicity	▪ Height<br>▪ Weight<br>▪ BMI<br>▪ BMI for age percentile (pediatric)<br>▪ Systolic blood pressure<br>▪ Diastolic blood pressure	▪ oSDB/OSA diagnosis<br>▪ CPAP<br>▪ Sleep study data<br>▪ Hypertension (incl. meds)<br>▪ Coronary artery disease<br>▪ Stroke<br>▪ Diabetes mellitus<br>▪ Allergies<br>▪ Asthma<br>▪ Lung disease<br>▪ Craniofacial<br>▪ Syndrome<br>▪ Upper airway infections<br>▪ Other diseases	▪ SDB/OSA (household)<br>▪ Hypertension<br>▪ Lung disease<br>▪ Other diseases<br>▪ Parental education	▪ Breastfeeding (pediatric)<br>▪ Tobacco smoke exposure<br>▪ Previous surgery

[1]For around half of pediatric patients, we also had data on the following variables from the Pediatric Sleep Questionnaire based on parental concern (yes/no): behavioral issues, poor academic performance, daytime sleepiness, mouth breathing, snoring, pause in breathing, gasping during sleep, nocturnal enuresis, swallowing problem, previous tonsillitis.

Table S2. Sleep study or polysomnography (PSG) parameters

Parameter	Description
Respiratory events	
• Apnea-hypopnea index (AHI)	Number of apnea/hypopnea/mixed apnea events per hour of sleep for the following stages and positions: overall, non-supine, supine, REM, NREM. For obstructive events, the Obstructive AHI or OAHI is reported.
• Respiratory disturbance index (RDI)	Average number of apneas, hypopneas and RERA events per hour of sleep (adult). The average number of apneas, hypopneas, and RERA events per hour of sleep (similar to AHI but includes arousal-related events)
• Arousal index	# minor awakenings (sleep lasting 3 to 15 seconds) per hour of sleep. Includes spontaneous types of arousal, also called spontaneous arousal index.
• Arousal thresholds	Events that can cause awakenings from sleep (adult). Arousals are important in airflow or blood-gas disturbances that may occur during sleep. The respiratory arousal index and spontaneous arousal index are often used as surrogate indicators of arousal thresholds
• Respiratory effort related arousal (RERA)	Events where partial flow limitation is seen and there is an associated arousal noted by EEG criteria indicating fragmentation of sleep, but no associated oxygen desaturation is present (adult)
• Isolated obstructive apnea /mixed apnea index	# of obstructive apneas and mixed apneas per hour of sleep
• Central apnea	# of central apneas (adult)
• Central apnea index (CAI)	# of central apneas per hour of sleep (central apnea = no airflow and no respiratory effort)
• Central apnea/hypopnea index (CAHI)	# of central apneas/central hypopneas per hour of sleep
OSA severity	AHI: mild=5-15, moderate=15-30, severe>30<br>OAHI: mild=1.5-5, moderate=5.1-10, severe>10
Sleep architecture	Characterization of the different amount of time spent in each stage of sleep during the night
• Sleep efficiency	Total sleep time divided by total recording time
• % Stage N1 sleep	An elevated amount of stage N1 sleep indicates sleep fragmentation
• % Stage R sleep	REM sleep is preferential to the early morning hours and since hypotonia occurs during this stage, the risk of OSA is higher
• % supine sleep	OSA can be positional in nature. In the supine position, the tongue is more prone to prolapse posteriorly and obstruct the airway
• % REM supine sleep	REM supine sleep is the condition when OSA would be predicted to be the worst especially for a child with obesity and DS
Oxygenation and ventilation	
• Oxygen nadir	Lowest oxygen saturation at specified time (total, awake, asleep)
• % time spent ≤90% O_2	Percentage of time spent with arterial oxygen saturation at or below 90% (pediatric)
• % time spent ≤88% O_2	Percentage of time spent with arterial oxygen saturation at or below 88% (adult)
• Mean SpO_2	Average oxygen saturation for three sleep stages (awake, asleep, REM)
• SpO_2 distribution <90%	% of time with oxygen saturation <90%
• 3% O_2 desaturation index	# of events/hour where there was a 3% oxygen desaturation from baseline (pediatric)

• % CO_2 distribution>45mmHg	% of time with CO_2 >45 mmHg (pediatric)
• % CO_2 distribution>50mmHg	% of time with CO_2 >50 mmHg (pediatric)
• Mean $EtCO_2$	Average end-tidal CO_2 (pediatric)
• Peak P_{CO2}	Highest CO_2 measurement (pediatric)
• 90% Max $EtCO_2$	Maximum end-tidal CO_2 exhaled; assesses ventilation (pediatric)
Mean heart rate	Average heart rate while asleep
Nadir heart rate	Lowest heart rate while asleep
PLM index	# of periodic limb movements/hour while asleep

Table S3. Characteristics of 16 children with oSDB and mRNA-seq data according to EBV± [1]

Variable	EBV+	EBV-	*p*-value
n (%)	7 (43.75)	9 (56.25)	--
Average age (median)	8.88 (9.52)	7.85 (7.38)	t-test: 0.35
n female (%)	2 (28.57)	6 (66.67)	Fisher's exact: 0.98
n non-White, Latino or mixed (%)	5 (71.43)	2 (22.22)	Fisher's exact: 0.07
Average height, in cm (median)	131.17 (132.80)	126.02 (122.00)	t-test: 0.48
Average weight, in kg (median)	46.52 (35.50)	35.50 (25.85)	t-test: 0.46
Average BMI (median)	23.28 (23.35)	24.53 (16.50)	t-test: 0.42
Average BMI for age (median)	84.23 (97.80)	69.92 (67.00)	t-test: 0.23
Average systolic blood pressure (median)	113.88 (112.00)	112.00 (111.00)	t-test: 0.24
Average diastolic blood pressure (median)	72.52 (74.00)	74.00 (71.00)	t-test: 0.46
n breastfed (%)	5 (71.43)	6 (66.67)	Fisher's exact: 0.63
n tobacco exposure (%)	1 (14.29)	2 (25.00)	Fisher's exact: 0.88
n allergies (%)	3 (42.86)	6 (66.67)	Fisher's exact: 0.93
n asthma (%)	2 (28.57)	1 (11.11)	Fisher's exact: 0.40
n family history of oSDB (%)	3 (42.86)	5 (55.56)	Fisher's exact: 0.84
n family history of hypertension (%)	3 (42.86)	3 (33.33)	Fisher's exact: 0.55
n family history of diabetes (%)	3 (42.86)	4 (44.44)	Fisher's exact: 0.71
n poor academic performance [2] (%)	2 (28.57)	1 (11.11)	Fisher's exact: 0.40
n daytime sleepiness [2] (%)	6 (85.71)	5 (55.56)	Fisher's exact: 0.23
n mouth breathing [2] (%)	4 (57.14)	5 (55.56)	Fisher's exact: 0.67
n gasping during sleep [2] (%)	2 (28.57)	4 (50.00)	Fisher's exact: 0.92
n swallowing difficulty [2] (%)	1 (14.29)	2 (25.00)	Fisher's exact: 0.88
Average sample RIN (median)	7.16 (7)	7.09 (7)	t-test: 0.43

[1] Only three children were prescribed with CPAP, resulting in small numbers per variable in the CPAP group.

[2] Variables from the Pediatric Sleep Questionnaire.

Table S4. Kyoto Encyclopedia of Genes and Genomes (KEGG) pathways that are enriched within the gene network connecting 12 differentially expressed genes in pediatric tonsils

Pathway	Total genes within pathway	Unadjusted -*p*	False discovery rate adjusted-*p*
Wnt signaling pathway	158	7.54e-42	2.4e-39
Pathways in cancer	530	1.4e-26	2.22e-24
Breast cancer	147	1.77e-17	1.88e-15
Hippo signaling pathway	154	7.36e-16	5.85e-14
Basal cell carcinoma	63	6.38e-14	4.06e-12
Melanogenesis	101	5.74e-11	3.04e-09
HTLV-I infection	219	7.54e-11	3.43e-09
Cell cycle	124	9.43e-10	3.75e-08
Proteoglycans in cancer	201	9.71e-09	3.43e-07
Transcriptional misregulation in cancer	186	2.47e-08	7.85e-07
Signaling pathways regulating pluripotency of stem cells	139	3.93e-08	1.14e-06
Chronic myeloid leukemia	76	6.22e-08	1.56e-06
Prostate cancer	97	6.38e-08	1.56e-06
Colorectal cancer	86	2.06e-07	4.69e-06
Adherens junction	72	4.62e-07	9.8e-06
Cellular senescence	160	1.5e-06	2.98e-05
Notch signaling pathway	48	3.23e-06	6.05e-05
cAMP signaling pathway	212	5.14e-06	9.07e-05
mTOR signaling pathway	153	6.36e-06	0.000107
Kaposi's sarcoma-associated herpesvirus infection	186	7.25e-06	0.000115
Hepatitis B	163	1.17e-05	0.000177
Insulin resistance	108	1.42e-05	0.000199
Focal adhesion	199	1.44e-05	0.000199
Viral carcinogenesis	201	1.6e-05	0.000211
Thyroid hormone signaling pathway	116	2.53e-05	0.000309
Mitophagy - animal	65	2.53e-05	0.000309
Acute myeloid leukemia	66	2.8e-05	0.00033
TGF-beta signaling pathway	92	3.19e-05	0.000362
Hedgehog signaling pathway	47	3.7e-05	0.000406
Endocrine resistance	98	5.05e-05	0.000535
Epstein-Barr virus infection	201	8.15e-05	0.000836
Thyroid cancer	37	0.000129	0.00128
Bladder cancer	41	0.000213	0.00205
Small cell lung cancer	93	0.000252	0.00236
Amphetamine addiction	68	3e-04	0.00272
Oxytocin signaling pathway	153	0.00107	0.00931
Endometrial cancer	58	0.00108	0.00931
Starch and sucrose metabolism	36	0.00135	0.0113
Osteoclast differentiation	128	0.00171	0.0139
Tight junction	170	0.0021	0.0167
MicroRNAs in cancer	299	0.00234	0.0181
Insulin signaling pathway	137	0.00252	0.0191
Measles	138	0.00263	0.0193
Glucagon signaling pathway	103	0.00267	0.0193
Melanoma	72	0.00285	0.0201
Th17 cell differentiation	107	0.00323	0.0224

Pancreatic cancer	75	0.0034	0.023
Leukocyte transendothelial migration	112	0.00405	0.0269
Hepatitis C	155	0.005	0.032
Maturity onset diabetes of the young	26	0.00503	0.032
AMPK signaling pathway	120	0.00568	0.0354

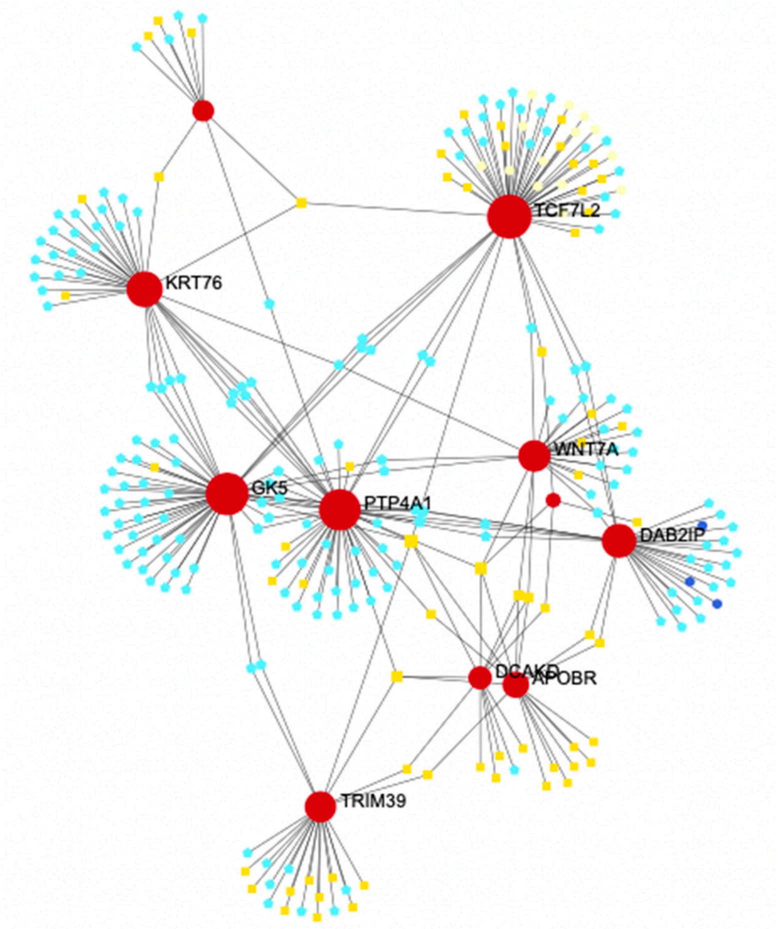

Figure S1. Protein-protein interaction-transcription factor-miRNA network connecting 12 significant DEGs from Table 1.
